# Occupational Exposure to Poorly Soluble Low Toxicity Particles and Cardiac Disease: A Look at Carbon Black and Titanium Dioxide

**DOI:** 10.3389/fpubh.2022.909136

**Published:** 2022-07-29

**Authors:** Robert J. McCunney, Mei Yong, David B. Warheit, Peter Morfeld

**Affiliations:** ^1^Brigham and Women's Hospital and Harvard Medical School, Boston, MA, United States; ^2^MY EpiConsulting, Duesseldorf, Germany; ^3^Consulting, Wilmington, DE, United States; ^4^Institute and Policlinic for Occupational Medicine, Environmental Medicine and Prevention Research of Cologne University Hospital, Cologne, Germany

**Keywords:** PSLTs, soluble particles, cardiac disease, carbon black, titanium dioxide

## Abstract

Environmental particulate exposure and the potential risk to people with various types of cardiac diseases, most notably cardiovascular disease, have aroused scientific and regulatory interest worldwide. Epidemiological studies have shown associations between exposure to airborne environmental particulate matter (PM) and mortality from cardiovascular disease (CVD). The associations reported, however, are complex and may not involve a direct role for PM, since air pollutants are diverse and highly correlated. This study examines the potential role of occupational exposure to two types of particles, namely, manufactured carbon black (CB) and titanium dioxide (TiO_2_), on the risk of cardiovascular disease. To address the risk of cardiovascular disease from exposure to carbon black and titanium dioxide, as reflective of poorly soluble low toxicity particles, we reviewed the published cohort mortality studies of occupational exposure to carbon black and titanium dioxide. Mortality studies of carbon black have been conducted in the United States, Germany, and the United Kingdom. Five mortality studies related to workers involved in the manufacture of titanium dioxide in the United States and Europe have also been conducted. In addition, a meta-analysis of the three-carbon black mortality studies was performed. In the random-effects meta-analysis, full cohort meta-SMRs were 1.01 (95% confidence interval (CI): 0.79–1.29) for heart disease; 1.02 (95% CI: 0.80–1.30) for ischemic heart disease; and 1.08 (95% CI: 0.74–1.59) for acute myocardial infarction (AMI) mortality. A small but imprecise increased AMI mortality risk was suggested for cumulative exposure by a meta-HR = 1.10 per 100 mg/m^3^-years (95% CI: 0.92–1.31) but not for lugged exposures, that is, for recent exposures. Results of five cohort mortality studies of titanium dioxide workers in the United States and Europe showed no excess in all heart disease or cardiovascular disease. In the most recent study in the United States, an internal analysis, that is, within the cohort itself, with no lag time, showed that the exposure group 15–35 mg/m^3^-years yielded a significantly increased risk for heart disease; however, there was no evidence of increasing risk with increasing exposure for any of the exposure categories. In contrast to environmental studies, the results of cohort mortality studies do not demonstrate that airborne occupational exposure to carbon black and titanium dioxide particulates increases cardiovascular disease mortality. The lack of a relationship between carbon black and titanium dioxide and CVD mortality suggests that the associations reported in air pollution studies may not be driven by the particulate component.

## Introduction

Cardiac disease, most notably cardiovascular disease (CVD), is one of the major causes of death worldwide. According to recent Centers for Disease Control data, CVD accounts for about 659,000 deaths per year in the United States (2019 figures). Epidemiological studies of environmental exposure to airborne particulates have reported associations with a variety of cardiovascular effects, including myocardial infarction, ischemic heart disease, and the major types of CVD (1–3). These effects were first reported among North American and European populations; an analysis of four Chinese studies generated similar results (4). Major risk factors for CVD include smoking, hypertension, high cholesterol, diabetes, family history, and obesity (5).

In light of the potential for environmental particulates to increase the risk of CVD, the American Heart Association published a position paper on particulate matter and heart disease and noted the following: “*It is the opinion of the writing group that the overall evidence is consistent with the causal relationship between PM*_2.5_ *exposure and cardiovascular morbidity and mortality*” (6). The European Society of Cardiology has drawn similar conclusions: “*There is abundant evidence that air pollution contributes to the risk of cardiovascular disease*” (7). The European Society further noted that “*Research should explore optimal methods of air pollution reduction and document the effects of air pollution on the incidence of cardiovascular disease and related mortality to motivate policymakers to intensify legislative efforts on air pollution production*” (7).

In light of these policy statements by professional groups of cardiology specialists in the United States and Europe as well as the results of *environmental* epidemiological studies, this study explores potential relationships between *occupational* exposure to carbon black (CB) and titanium dioxide (TiO_2_) regarding the risk of cardiac disease. The purpose of this study was to review the standardized mortality ratios (SMRs) related to cardiovascular disease from the published cohort mortality studies of occupational exposure to carbon black and titanium dioxide, two substances that are considered representative of a category of substances that are considered poorly soluble low-toxicity particles (PSLTs).

### Background: Carbon Black and Titanium Dioxide

**Figure 1:**
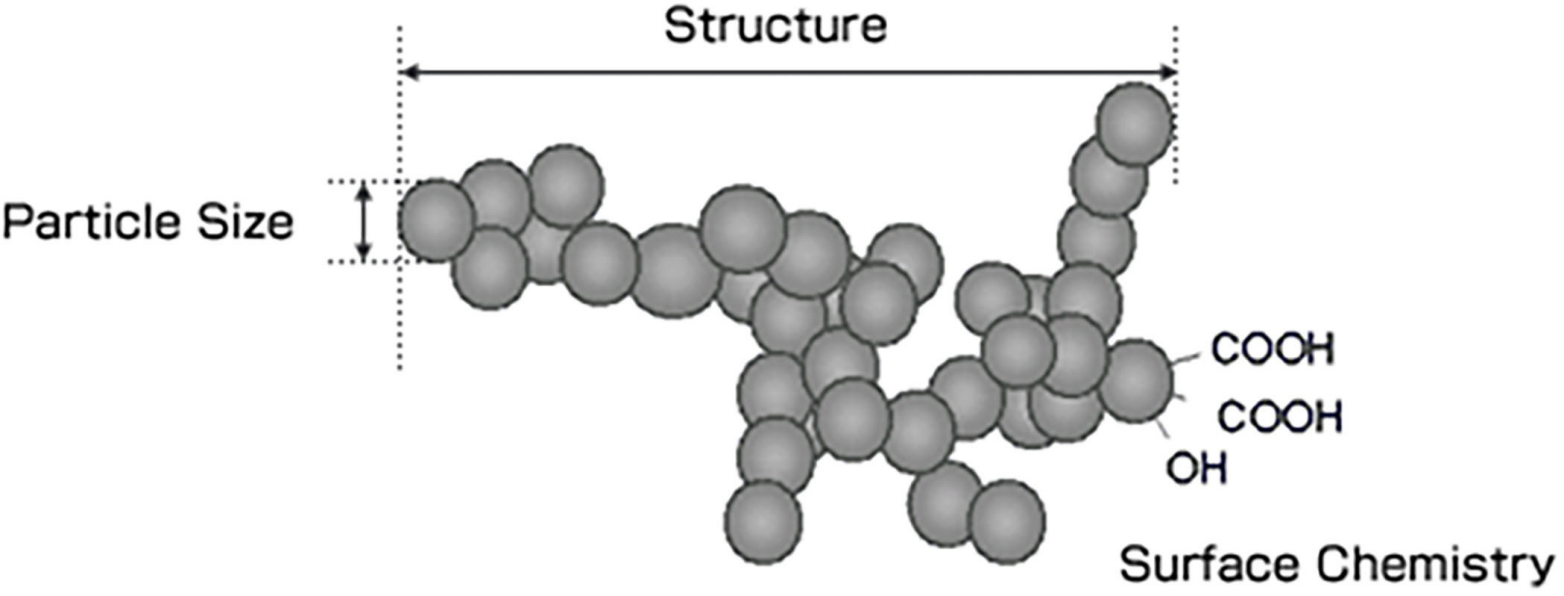
Structure of carbon black.

Carbon black is essentially pure carbon at 98%−99%. The material may also contain traces of polycyclic aromatic hydrocarbons that are *ad*sorbed and tightly bound on the surface of the particle. Carbon black's structure consists of fine particles that form tightly bound aggregates that then form agglomerates (refer to Figure 1).

There are 185 carbon black plants globally, with 17 in the European Union, 10 in Eastern Europe, 121 in Asia (81 of which are in China), 21 in North America, 7 in South America, and the remainder in the Mideast or Africa. Approximately 90% of all carbon black produced is used in the manufacturing of tyres and other rubber products, acting primarily as a reinforcing agent (8). The remaining 10% is used primarily in non-rubber industry production, such as black pigment in inks, coatings, and plastics, and to impart electrical conductivity to rubber and plastics.

More than 95% of carbon black worldwide is produced by the oil furnace process. Average primary particle diameters of furnace blacks range from 17 to 70 nm and those of thermal blacks from 150 to 500 nm (refer to Table 1). Since primary particles fuse to form aggregates, aggregates are the discrete, dispersible units that constitute the fundamental carbon core of carbon black particles. Clusters of aggregates may form and are known as agglomerates. Carbon blacks are usually highly agglomerated, with 10–1,000 particle aggregates per agglomerate.

**Table 1:** Typical ranges of properties for the five principal types of commercially produced carbon blacks^*^.

**Type of carbon black**	**Acetylene black**	**Furnace black**	**Gas black/channel black**	**Lampblack**	**Thermal black**
Average aggregate diameter	Not reported	~80–500 nm	Not reported	Not reported	300–810 nm
Average primary particle diameter (nm)	45–50	17–70	13–29	50–100	150–500
Surface area (m^2^/g)	60–70	20–300	90–320	20–95	6–15
Carbon (%)	99.8	97.3–99.3	Not reported	Not reported	99.4

* *Table abstracted from Table 89.3 of McCunney et al. “Carbon Black,” in Pattys Industrial Hygiene and Toxicology (2022, in press). Data are based on original material from M.-J. Wang, C. A. Gray, S. A. Reznek, K. Mahmud, and Y. Kutsovsky, Carbon black, in Kirk-Othmer Encyclopedia of Chemical Technology, 2003, and G. Locati, A. Fantuzzi, G. Consonni, I. Li Gotti, and G. Bonomi, Identification of polycyclic aromatic hydrocarbons in carbon black with reference to carcinogenic risk in tire production. Am. Ind. Hyg. Assoc. J. **40**(7), 644–652 (1979)*.

**Figure 2:**
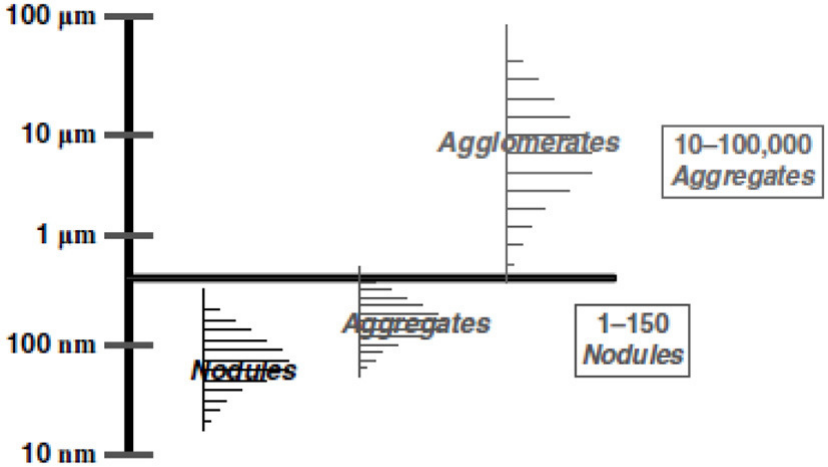
Size distributions of carbon black structural entities^*^. Abstracted from McCunney et al. Pattys (in press) original figure in Pattys courtesy of Cabot Corporation.

Figure 2 shows size distribution data for primary particles and aggregates of carbon black; neither primary particles nor aggregates, however, are generally available under typical settings of its manufacture or use. Complex agglomerates are the predominant carbon black structure outside the environment of the enclosed reaction chamber in which carbon black is manufactured. Agglomerate sizes range from the hundreds of nanometers to the hundreds of microns, which is the typical form of industrial produced and used carbon black. Primary particles, with sizes in the nanometer range, however, can be present in carbon black, but the commercial form of carbon black rarely includes primary particles because of their rapid transformation into aggregates and agglomerates. As a result, carbon black does not meet the EU definition of a nanomaterial, that is, “*a natural, incidental or manufactured material containing particles, in an unbound state or as an aggregate or as an agglomerate and where, for 50% or more of the particles in the number size distribution, one or more external dimensions is in the size range 1 nm*−*100 nm*.”

Titanium dioxide, a white organic compound has been used for many years in a vast number of diverse products to heighten the whiteness and brightness of materials. It has reflective characteristics and is known for being whitest and brightest of known pigments Metallic titanium and titanium dioxide forms are insoluble and unreactive, devoid of acute toxicity although chronic overload inhalation exposure at high concentrations of TiO_2_ in rat studies can increase risk of lung tumors (9). With the expansion of nanotechnology, the TiO_2_ material has been engineered into a variety of shapes and sizes, which has led to significant particle size reduction; this reduction of the particle size can lead to increased surface area that may increase toxicity.

## Methods

For this analysis, we reviewed the published occupational cohort mortality studies on carbon black and titanium dioxide in which death rates associated with various types of cardiac disease, including cardiovascular disease, have been evaluated. Cohort mortality studies have been conducted on occupational exposure to carbon black in the United States, Germany, and the United Kingdom (10–13). In addition, a meta-analysis of the three cohort mortality studies of carbon black workers has also been performed (12). Titanium dioxide cohort mortality studies that included assessments of cardiac disease risk have been conducted in the United States and Europe (13–15 and 2010, and 16). A meta-analysis of the cohort mortality studies of titanium dioxide reported no significant risk of all-cause or lung cancer mortality but did not address the risk of cardiac disease (14).

## Results

### Carbon Black: United States Cohort Mortality Study

The largest mortality study of carbon black workers conducted to date was performed in the United States and included 6,634 workers (10). Some of the workers began employment in the industry as far back as the 1930s. In this study, cumulative inhalable carbon black was assessed by individual lifetime exposure for all cohort members, with the metric being mg/m^3^-years. The study had a 98.5% ascertainment of vital status.

In the US cohort mortality study, 616 deaths occurred due to heart diseases (ICD-9 410–429), whereas 790 were expected. These results led to an SMR of 0.78 (95% confidence interval (CI): 0.72–0.84) in the full cohort (10). An evaluation of an inception cohort, that is a group of workers who were followed from the *inception*, i.e., the beginning, of their employment in the carbon black industry, was also performed. Among this group, 332 deaths from ischemic heart disease and the major type of cardiovascular disease occurred, whereas 394 were expected. The SMR was 0.84 (95% CI: 0.75–0.94; refer to Table 2).

**Table 2:** Cardiac mortality in US cohort.

	**Full Cohort** ***N*** **=** **6,634**				**Inception Cohort**. ***N*** **=** **3,890**			
	**Obs**	**Exp**	**SMR**	**95% CI**	**Obs**	**Exp**	**SMR**	**95% CI**
Diseases of the heart ICD-9 410-429	616	790	0.78	0.72-0.84	332	394	0.84	0.75-0.94
								
Ischemic Heart Disease ICD-9 410-414	272	309	0.82	0.75-0.90	272	309	0.88	0.78-0.99

Ischemic heart disease in the full cohort accounted for 511 deaths, whereas 622 were expected. The SMR was 0.82 (95% CI: 0.75–0.90). An evaluation of an inception cohort yielded 272 deaths, whereas 309 were expected. The SMR was 0.88 (95% CI: 0.78–0.99). In summary, a cohort mortality study of the largest cohort of carbon black workers showed no increase in the risk of death from diseases of the heart or ischemic heart disease (refer to Table 2).

### Carbon Black: United Kingdom Cohort Mortality Study

A cohort mortality study was conducted among five plants in the United Kingdom that included 1,147 workers employed over the period of 1951–1996 (13). In this analysis, the SMR of diseases of the circulatory system (ICD 8: 390–458) was 1.0 based on 157 deaths (95% CI: 0.85–1.17). This cohort was later updated to assess the risk of lung cancer (15) and further updated in a meta-analysis of all three carbon black cohorts in which additional deaths from heart disease were tabulated and analyzed (12). In this analysis, all of the ICD codes used in the respective mortality studies for “all heart disease” were harmonized and divided into acute myocardial infarctions and the category “all heart disease.”

### Carbon Black: German Cohort Mortality Study

A cohort mortality study of 332 deaths at the largest carbon black plant in Europe showed a SMR of 1.26 based on 103 observed deaths of heart disease [ICD-9: 410–429; 95% CI: 1.03–1.53; (11)]. German national rates were used as the reference population. Workers who smoked more than 24 cigarettes per day had the highest relative risk of heart disease (7.50; 95% CI: 1.53–36.68).

An inception cohort was also evaluated. Ischemic heart disease was significantly elevated: SMR 1.30 based on 75 deaths (95% CI: 1.02–1.63). Other heart disease ICD codes 415–429 indicated non-statistically significant excesses of SMR 1.28 in the full cohort and 1.47 in the inception cohort. These latter results were based on the reference population of West Germany.

The findings were slightly different in an analysis that used the German State (North Rhine-Westphalia), where the carbon black factory was located, as the reference population. North Rhine-Westphalia was used as a more appropriate reference group because of the high rate of smoking in this area. SMRs for heart disease, ischemic heart disease, and other heart diseases were all non-statistically significantly elevated (refer to Tables 3, 4; 17).

**Table 3:** Cardiac mortality in German cohort: reference population: West Germany (from 17).

	**Full Cohort**. ***N*** **=** **1,535**			**Inception Cohort**. ***N*** **=** **1,276**		
	**Obs**	**SMR**	**95% CI**	**Obs**	**SMR**	**95% CI**
Diseases of the heart ICD−9: 410–429	103	1.29	1.05–1.57	60	1.39	1.06–1.79
Ischemic Heart Disease ICD−9: 410–414	75	1.30	1.02–1.63	43	1.36	0.98–1.83
Other Heart Disease ICD−9 415–429	28	1.28	0.85–1.85	17	1.47	0.86–2.35

**Table 4:** Cardiac mortality in German cohort: reference population: North Rhine–Westphalia.

	**Full Cohort**. ***N*** **=** **1535**			**Inception Cohort**. ***N*** **=** **1,276**		
	**Obs**	**SMR**	**95% CI**	**Obs**	**SMR**	**95% CI**
Diseases of the heart ICD−9: 410–429	103	1.17	0.96–1.42	60	1.28	0.98–1.65
Ischemic Heart Disease ICD−9: 410–414	75	1.19	0.94–1.49	43	1.27	0.92–1.71
Other Heart Disease ICD−9 415–429	28	1.13	0.75–1.63	17	1.31	0.76–2.10

In summary, cohort mortality studies of more than 9,000 carbon black workers in the United States, United Kingdom, and Germany indicated the following:

- No excess in cardiac-related mortality was detected in the UK and US cohorts.
- In the German cohort, the cardiac excess detected was of borderline significance when German national death rates were used as the reference population but not when state rates, most notably, those of North Rhine-Westphalia were used as the reference population (16).

In follow-up to these three studies, a meta-analysis was conducted to determine whether there was an elevated risk of cardiac disease in carbon black workers as reported in the three cohort mortality studies described earlier (12). To conduct the meta-analysis, it was necessary to combine standardized mortality ratios and Cox proportional hazard results from the US, UK, and German carbon black production workers. Fixed and random-effects analyses were performed. The analysis addressed mortality for heart disease, ischemic heart disease, and acute myocardial infarction. The results for the full cohort random-effects SMRs were 1.01 (95% CI: 0.79–1.29) for heart disease, 1.02 (95% CI: 0.8–1.30) for ischemic heart disease, and 1.08 (95% CI: 0.74–1.59) for acute myocardial infarction [refer to Table 5; (12)].

**Table 5:** Meta–analysis of US, UK, and German cohort mortality studies.

**Cardiac Disease**	**SMR**	**95% Confidence Intervals**
Heart disease	1.01	0.79–1.29
Ischemic heart disease	1.02	0.80–1.30
Acute myocardial infarction	1.08	0.74–1.59

The authors addressed the highest SMR found for acute myocardial infarction (AMI) [see Tables 11, 12 in (12)]. Random-effects hazard ratios for AMI in an internal analysis indicated a relative risk of 1.10 (95% CI: 0.92–1.31) for cumulative exposure based on 432 deaths among the three cohorts. An internal analysis of an inception cohort that included 138 observed cases showed a relative risk of acute myocardial infarction based on a cumulative of 1.05 (95% CI: 0.97–1.13).

### Titanium Dioxide (TiO_2_) Cohort Mortality Studies

There have been five published reports of cohort mortality studies conducted among workers involved in the manufacturing of titanium dioxide in the United States and Europe (17–21).

In the first cohort mortality study of titanium dioxide workers in the United States, 1,576 workers exposed to TiO_2_ were observed from 1956 to 1985 at two DuPont TiO_2_ production plants in the United States (17). Diseases of the circulatory system accounted for 107 deaths, whereas 115.5 were expected. Ischemic heart disease accounted for 76 deaths, whereas 81.5 were expected (refer to Table 6). The standardized mortality ratios (SMR) showed no excess in all heart disease or ischemic heart disease. The results were based on both US and DuPont employee reference rates. As noted in Table 6, the results showed no statistically significant elevation in the categories “all heart diseases” or “ischemic heart diseases,” while larger but non-significant SMRs resulted from a comparison with the mortality rates of DuPont employees. Note that the figures for the number of deaths when the respective US and DuPont reference rates were used were taken directly from the published report. Chen and Fayerweather did not describe the basis of the discrepancy. Nonetheless, the differences are negligible and do not affect the overall conclusion of the study.

**Table 6:** US cohort mortality study of DuPont TiO_2_ production workers (17).

**1576 participants**	**Number of deaths**	**SMR**	**90% CI**	**Reference rates**
All heart diseases	107	0.93	0.80–1.09	US
Ischemic heart diseases	76	0.93	0.78–1.13	US
All heart diseases	102	1.15	0.98–1.40	DuPont
Ischemic heart diseases	73	1.09	0.90–1.35	DuPont

#### US Study on TiO_2_

A retrospective cohort mortality study was conducted among 4,241 US TiO_2_ workers who were employed for at least 6 months, on or after 1 January 1960, at four TiO_2_ plants in the United States (18). Among 171 deaths,^1^ in comparison to US disease rates, the SMR was 0.9 (95% CI: 0.7–1.0). These results indicate that there was no increase in mortality from all heart diseases. In an external analysis, there was no dose-response relationship shown between three categories of exposure (low, medium, and high) in relation to the category “all heart disease.” No significant association was observed with increasing cumulative exposure (*p*-value of 0.10; refer to Table 7).

**Table 7:** Internal comparisons for all heart disease (18).

**Fryzek et al., 14**				
**Cum. Expo**.	**Number of deaths**	**HR**	**95% CI**	**Adjustments**
Low	22	1		Date of hire, age, sex, and geographic are
Medium	57	1.1	0.7–1.8	
High	44	0.8	0.5–1.4	

#### European Cohort Mortality Study of TiO_2_ Workers

A mortality follow-up study of 15,017 workers (14,331 men) employed in 11 factories producing TiO_2_ in 6 European countries [Finland, France, Germany, Italy, Norway, and the United Kingdom; (19)]. The start of follow-up varied by plant and ranged from 1950 to 1972, and the end of follow-up ranged from 1997 to 2001. During the follow-up, 2,652 cohort members died.

Country-specific SMRs were calculated using national mortality rates and pooled across countries using a Poisson model. Therefore, the expected numbers of deaths are not the integral numbers.

The risk of ischemic heart disease mortality was statistically *decreased* significantly in men (refer to Table 8).

**Table 8:** European cohort study on TiO_2_.

**Boffetta et al., 16**	***N* = 15,017**				
**Ischemic heart disease**	**Sex**	**Number of deaths**	**SMR**	**95% CI**	**Reference rates**
ICD:410–414	Men	629	0.88	0.81–0.95	national
	women	5	0.63	0.20–1.41	national

Authors of another study in the United States defined a much-expanded cohort of DuPont TiO_2_ production workers including the oldest and largest facility studied by (17), with a longer follow-up period, and better exposure data. The first of these investigations studied the mortality of a cohort of 5,054 individuals (90% male, 81% white) employed at three DuPont TiO_2_ production facilities (20) evaluated cause-specific standardized mortality ratios (SMRs) and stratified the results by the plant for workers employed for at least 6 months between 1935 and 2005 (20). No excess mortality in all heart diseases was observed. The SMR was larger but not significant, in comparison with the mortality rates of DuPont employees (refer to Table 9).

**Table 9:** Results from extended follow–up of the DuPont cohort.

**Ellis et al., (20)**				
	**Number of deaths**	**SMR**	**95% CI**	**Reference rates**
All heart diseases	306	0.92	0.82–1.03	US
	305	1.04	0.93–1.16	DuPont

Following this study, a cohort of 3,607 workers employed in the same three DuPont titanium dioxide production facilities was followed from 1935 to 2006. This study examined exposure-response between mortality and cumulative exposure to TiO_2_ and TiCl_4_. The more restrictive entry criteria were applied in an effort to reduce the uncertainty in the exposure estimates.The cohort included 833 deaths. Like the earlier report, no excess in all heart disease was observed (15; refer to Table 10).

**Table 10:** US TiO_2_ workers at DuPont plants.

**Ellis et al., (21)**				
All heart diseases	519	0.82	0.75–0.89	US

In an internal analysis (Table 11) with no lag time, only the exposure group 15–35 mg/m^3^-year yielded a significantly increased risk for heart disease, while the relative risks of other 4 exposure categories were not significantly increased. When exposure was lagged 10 years, results were similar with the relative risk estimates similar or slightly higher for each exposure level.

**Table 11:** Internal analyses (21).

**Ellis et al. (21)**	**Lag** **=** **0 year**		**Lag** **=** **10 years**	
	**HR**	**95% CI**	**HR**	**95% CI**	**Adjustments**
< 5 mg/${\text{m}}_{-}^{2}$years	1		1		Age, gender, ethnicity, plant first employed, and calendar time
5–15	1.30	0.89–1.89	1.47	1.02–2.11	
15–35	1.61	1.13–2.31	1.65	1.16–2.34	
35–80	1.32	0.90–1.94	1.36	0.92–2.00	
80+	1.27	0.84–1.90	1.51	1.00–2.25	

The authors argued that these results were driven by the sub-cohort at Edgemoor plant (EM). Lagging exposure of 10 years would have the least effect on cumulative exposure for the EM cohort since it is the oldest. There was no evidence of an increasing risk of cardiac mortality with increasing exposure to titanium dioxide.

## Discussion

The meta-analysis of carbon-black workers included all three carbon-black cohort mortality studies published to date in the United States, the United Kingdom, and Germany. Thus, it has the greatest potential to identify cardiovascular disease risks among these carbon black workers. The meta-analytic procedures that were used to combine specific results of the three-carbon black cohort mortality studies led to enhanced statistical power to identify even small associations. In the analysis, SMR and Cox proportional hazard results focused on ischemic heart disease and acute myocardial infarction as the key components of cardiovascular disease. The availability of reasonably detailed employment histories and exposure assessments in the three cohorts allowed quantitative evaluation of the risk of cardiovascular mortality through the use of standardized individual carbon black exposure estimates. These meta SMRs were unexceptional and showed no association with the duration of exposure to carbon black. These results in a weight of the evidence assessment indicate that carbon black exposure does not increase cardiac disease mortality.

A weight of the evidence assessment of titanium dioxide cohort mortality studies of workers in the United States and Europe also found no significant increase in the risk of cardiovascular disease. We conclude that occupational exposures to carbon black and titanium dioxide indicate no increase in the risk of death from cardiovascular disease.

The discordance of our results of occupational exposure to these particulates in contrast to environmental exposure to ambient pollution that includes particulates and other substances may reflect differences in particle size or that environmental exposures include many other substances, in addition to particulates.

## Author Contributions

All authors listed have made a substantial, direct, and intellectual contribution to the work and approved it for publication.

## Conflict of Interest

MY was employed by MY EpiConsulting. DW was employed by Consulting. RJM, MY and DW are consultants to the International Carbon Black Association. The remaining author declares that the research was conducted in the absence of any commercial or financial relationships that could be construed as a potential conflict of interest. The reviewer KM declared a past co-authorship/collaboration with the author MY to the handling editor.

## Publisher's Note

All claims expressed in this article are solely those of the authors and do not necessarily represent those of their affiliated organizations, or those of the publisher, the editors and the reviewers. Any product that may be evaluated in this article, or claim that may be made by its manufacturer, is not guaranteed or endorsed by the publisher.

## Footnotes

^1^*Note that the ICD codes were not specified in the article but are presumed to be ICD-9-410-414*.
